# Dr. Hughes’ Letter

**Published:** 1853-08

**Authors:** J. C. Hughes

**Affiliations:** New York


LETTER FROM DR. HUGHES.

The following letter, written by Prof. Hughes, a delegate from the college, is descriptive of the American Medical Association, and will be perused with interest. It is one of a series which we shall continue to publish in our future numbers:

New York, May 8th, 1853.

Gentlemen:—

Having written you some days since from the city of *brotherly love*, giving you a bird's eye view of the profession, men and things in *that* city, as well as Baltimore, and now having arrived in New York, which is of itself a miniature world, I will continue the subject.

That which will most interest you at this time will be the proceedings of the Medical Association, which has just closed its session, and of which in the course of my letter I shall make as full a report as my time will permit.

I left Philadelphia on the afternoon of the 2d inst., reaching this city same evening. Having stopped at the Irving House and taken supper, I wandered out on Broadway to have a view of that great thoroughfare. The day having rolled itself into oblivion, and having tested the truth of the poet when he said, "Tired nature's sweet restorer, balmy sleep," I was again ready for action.

This, the 3d inst., being the day appointed for the meeting of the American Medical Association in this city, I repaired at the hour appointed, with hundreds of others, to the Bleecker street Presbyterian Church, the place appointed for that purpose. After presenting myself at the Committee room and having received my certificate of membership I took my seat, and at 11 o'clock the Association was organized, Beverly R. Wellford in the chair. The President congratulated the members of the Association upon the happy return of its sixth anniversary, the thronged attendance of delegates from all parts of the Union, and the flattering prospects and advancement of the profession generally.

Dr. F. Campbell Stewart, Chairman of the Committee of Aarrangements and reception, rose and congratulated the Association upon the recurrence of its anniversary, and after some very appropriate remarks upon the object of the Association, and the interest manifested in behalf of it by the members of the profession in this city and throughout the State, closed by tendering the Association, in the name of the united profession of New York, a sincere, hearty and united welcome.

The names of the delegates having been called, the number present over four hundred, and nearly every State in the Union represented.

On motion, a recess of fifteen minutes was taken, to allow the delegates to select one of their number from each State, as a committee to nominate officers for the ensuing year, after which Dr. Pope extended an invitation to the Association to hold its next annual meeting at St. Louis. This was responded to by Dr. Hays, of Philadelphia, in a manner worthy the position he occupies, and gratifying to every western delegate, and I might say to every member present, as the vote when taken was unanimous for St. Louis.

Dr. Condie, of Philadelphia, chairman of the committee of publication, reported upon the publication and sale of the different volumes of the transactions of the Association, and urged an increase of assessment upon each member from three to five dollars, as the former was insufficient to publish a volume of such vast importance. This resolution was unanimously adopted.

Dr. Stewart presented a report recommending the admission of Dr. Marshall Hall, of London; Surgeon Mower, U. S. A., and others, to participate in the proceedings by initiation. A committee was appointed to wait upon Dr. Hall, but he had not yet arrived, and from some cause was unable to reach the city during the session of the Association.

The President, Dr. Wellford, now being called upon, made a very appropriate and eloquent address. He spoke at length upon the objects of the Association, the good to be accomplished, its stimulus to the profession, its influence upon medical colleges, its effect upon State as well as national legislation, in the prevention of adulterated drugs, showing the amount of different articles that had, during the past year, been rejected, urged strongly the establishment of State and County Societies, the impropriety of tolerating any one as a practitioner who chooses to dub himself doctor of the healing art, and closed with a touching tribute to the memory of Drs. Drake and Horner.

After the thanks of the Association were presented to the President for his able address and a copy requested for publication, the Secretary read a resolution passed by the medical Society of Virginia, recommending the appointment of a chemist to analyze the most prominent nostrums of the day, and publish the result monthly in the leading newspaper of each State. Also a communication from the American Medical Society in Paris, appointing Drs. Pittman. Walton and McIlvaine, to attend this Association.

On motion of Dr. Atlee, the committee of publication was directed to send a full set of the transactions of the Association to the Society at Paris.

The committee on nominations reported the following officers for the ensuing year:

*For President,* Dr. Jonathan Knight, of Connecticut.

The announcement of Dr. Knight's name as President was very unexpected to himself, as well as most of the Association. It has been customary, as you are aware, to elect from the city or State in which the Association meets; this rule was varied from in only one instance since the organization of the Society, and that was in Baltimore. This, as we all know, is a bad precedent to establish. The effect would be, that a man, however prominent he might be in the profession, and however qualified to occupy the position, unless fortunate enough to reside in the city or State in which the Society met, would never be promoted.

I am not certain (though a member of the committee myself) that the established usage even in this instance would have been deviated from, had it not been that the New York delegation was not unanimous upon its own selection. Dr. Francis' name was presented to the committee as the one selected, but there seemed to be much feeling in favor of Dr. Mott, and I might say that this was the sense of a majority of the committee. And as there was now an opportunity of changing the precedent heretofore established, the committee at once united upon Dr. Knight, a gentleman of the highest attainments in his profession, and thoroughly acquainted with parliamentary usages. The nomination met with the hearty co-operation and sanction of the Association.

*Vice Presidents,* Drs. Usher Parsons, R. I., Lewis Condict, N. J., Henry R. Frost, S. C., and R. L. Howard, Ohio. These nominations being made by the committees from the Eastern, Middle, Southern and Western States, my nomination of Dr. Howard from the Western States was unanimously adopted.

*Secretaries,* Edward L. Beadle, N. Y., and E. L. Lemoine, Mo.

*Treasurer,* Dr. Francis Condie, Penn.

Drs. Good, Watson and Atlee, were appointed a committee to conduct the President elect and other officers to their seats.

Dr. Knight, on taking the chair, made some very appropriate remarks, returning his thanks to the Association for the honor conferred upon him.

A vote of thanks to the late President, Dr. Wellford, for his dignified, courteous and efficient manner in the chair, was by 'rising vote' carried unanimously. Also a vote of thanks to the retiring Secretary, Dr. Good—when the Society adjourned.

MAY 4th, 9 O'CLOCK A. M.—The Association met pursuant to adjournment, Dr. Knight in the chair. Then, after the reading and approval of the minutes of the previous day, nothing of importance came up, until the reports of standing committees were called for.

Dr. Meigs, of Philadelphia, presented a report on "Acute and Chronic Diseases of the Uterus," with a request that it should be referred to the committee of publication without reading, which report was adopted.

Dr. Condie, of Pa., Chairman of the committee on the causes of Tubercular Disease, finding the subject of so much importance, and fearing that he could not do it justice in the allotted time, asked a continuance of the committee, which was granted.

Dr. Emerson, of Philadelphia, read a synopsis of a report on the agency of refrigeration produced by the upward radiation of heat, as an exciting cause of disease.

Dr. Campbell, of Georgia, presented a report on typhoid fever, a synopsis of which was given verbally.

The above reports were both received and referred to the committee on publication.

Dr. John Atlee, chairman of the committee on epidemics of New Jersey, Pennsylvania, Delaware and Maryland, after making some humorous remarks, asked further time to report, which was granted.

Dr. Sutton, of Ky., made a report on the epidemics of Tennessee and Kentucky, a synopsis of which was read and referred to the committee on publication.

Dr. Pitcher, of Michigan, chairman of the committee on medical education, made a lengthy and able report, after which some resolutions upon this subject were submitted and adopted.

Dr. Joseph M. Smith, of New York, chairman of the committee on voluntary communications, reported fifteen received, and awarded the first prize of $100 to Dr. Waldo J. Burnett, of Boston, Mass., for his treatise on "The 'Cell,' its Physiology, Pathology and Philosophy,"

Another prize of $100 was awarded to Washington L. Atlee, for his treatise upon the "Surgical Treatment of Fibrous Tumors of the Uterus."

The remaining communications were highly spoken of, and some of them referred to the committe on publication, but no awards made.

Dr. March, of New York, having prepared an article on "Diseases of the Hip Joint," after having given a verbal abstract of same, was requested to read the article at length during recess, in the Crosby street Medical College. I did not have the pleasure of hearing this, but it was reported as an able document.

A resolution was offered by Dr. Atlee, of Pa., that a committee of five be appointed to solicit subscriptions from members of the Association for the purpose of procuring a suitable stone, with an appropriate inscription for insertion, in the name of the American Medical Association, in the National Monument to the Memory of Washington, now in progress of erection at Washington City. This met with a hearty reception, and many of the members at once paid over to the treasurer for that purpose.

After which several resolutions were offered upon appointing committees to memorialize the legislative bodies of each State upon the adoption of some course for the suppression of quackery.

After a recess, the Association was again called to order at 1½ o'clock. Over one hundred new delegates had taken their seats during the day. After some resolutions upon medical schools giving two courses of lectures the same year, a paper upon Morbid Growths of the Larynx was read by Dr. Buck, of N. Y., and on motion referred to the publishing committee.

Dr. Simons, chairman of the committee to memorialize Congress on having the medical statistics of the United States printed separately, for the use of the medical profession, reported that through Senator Butler, of South Carolina, the subject had been brought before the Senate and referred to the committee on commerce, and has not been heard of since; but as the Presidential election and other political excitements were now nearly at a close for a time, the committee hoped to be continued and now considered as reporting progress, which was granted.

Dr. Peasly, of N. H., offered a resolution on the subject of granting diplomas to those persons who intended practicing irregularly. This excited considerable discussion, but was finally laid upon the table—when the Society adjourned until nine o'clock Thursday morning.

The Society met pursuant to adjournment. The minutes of the previous day were read and approved. The names of a number of new delegates were announced.

After which Dr. Ziegler presented a preamble and resolutions touching the mode of organizing county and State Medical Societies whice was referred to the committee on that subject.

Dr. Davis, of Illinois, from the committee on Medical Literature, made a very elaborate and able report, which was read at length, and referred to the committee on publication.

The analyzation of quack medicines was again taken up, and created some discussion, and the resolutions offered were laid upon the table.

The report of the committee on Malignant Diseases, of which Dr. Gross was chairman, was presented by Dr, Yandall, Ky., of which an abctract was read, and referred to the committee on publication.

Several resolutions were again offered on the subject of Medical Colleges, requiring from their graduates certain pledges in behalf of the honor and dignity of the profession, and requiring from them a strict adherence to the code of ethics of this Association. This subject after some discussion, was referred to a committee, of which Dr. Stille was chairman, to report at the presnt session.

The committee made the following report:

*Resolved,* That, in order to preserve the purity and honor of the profession, it is recommended to the several medical colleges, and such other boards as are by law authorized to examine candidates for admission to the medical profession, to require from every graduate or licentiate his signature to the code of ethics; and they further recommend, that the formal administration of a pledge faithfully to observe and keep the same code to form part of the public exercises at medical commencements ; which, upon motion, was adopted.

After a vote of thanks to the profession of New York and the institutions which had received them so hospitably, also the President and Secretary, and the press, the Association adjourned *sine die.*

Before closing my letter, I must say a few words respecting the reception of members of the Association by the profession of New York. The committees of arrangements and reception were most untiring in their efforts; nothing was left undone that was calculated to make our visit a pleasant one.

On Tuesday morning cards of invitation were received by the delegates, inviting us to attend parties that evening, given by Gov. HAMILTON FISH, Drs. Isaac Wood, Willard Parker, G. P. Cammun and James R. Wood ; and on Wednesday evening, at ex-Mayor Kingsland, Drs. Edward Delafield, James Anderson, Wm. Detmold and

Isaac E. Taylor's. On Thursday evening, to a public dinner at Metropolitan Hall, and on Friday evening, at Drs. John Cheeseman's, John Watson, Horace Green and Lewis Tayre's. The parties given by these gentlemen were rich beyoud description, and to the bountifully spread tables justice was done, not Homeopathically, but Alopathically.

The dinner of Thursday evening baffles description. It was served in Metropolitan Hall, where over one thousand persons joined in the merry festival. The tables were magnificent, presenting the choicest viands, wines and delicacies, surpassing, "so says the press,' 'any thing that has ever been gotten up in New York.

After the company were seated, Dr. Parker made a few very appropriate remarks, and closed by bidding the guests a hearty welcome. Music, toasts, speeches, and the displacement of champagne corks, were the order of the evening; to which migh tbe added, smiles and greetings from the ladies who crowded the galleries.

Scarcely had the *echo* of the Metropolitan Hall festivities subsided, before some four hundred of us were aboard of the steamboat *Hero*, which plied from Pier No. 3, at 9 o'clock Friday morning, for the different institutions of interest to the profession, situated on Staten, Randall's, Ward's and Blakwelle's Islands. The day was very unpleasant, but the hiliarity that prevailed the evening before was partially kept up, and all went merry as a marriage bell. These institutions present much of interest to the physician. The Marine, or Quarantine Hospital on Staten Island, is devoted almost exclusively to the reception of contagious or infectious diseases, and is in charge of the Commissioners of Emigration, and is supported by the emigrant commutation fund. The number of patients in 1852 was 8,887.

*The Bellevue Hospital.*—This institution is under the supervision of the Governors of the Alms House, and under the professional charge of a medical Board, consisting of nine visiting Physicians and six Surgeons. Present number of patients, 570.

*The Emigrant Refuge, Hospital and Nurseries.*—This institution is on Ward's Island, and under the care of the Commissioners of Emigration, appropriated exclusively for sick and destitute aliens, who have landed at this port within five years. The total number cared for and treated in 1852, (including 523 births,) was 15,182. It is supported in the same manner as the Quarantine Hospital.

*The Nurseries on Randall's Island.*—This institution is under the care of the Governors of the Alms House, and devoted to the support and education of destitute children. The number taken care of in 1852 was 3,213. The present number of inmates is about 1,300.

*The Seaman's Retreat.*—This institution is on Staten Island, under charge of a Board of Trustees, and supported by a tax on sailors. The number of inmates varies from 200 to 300.

*Sailor's Snug Harbor.*—This is for the support of infirm and superannuated seamen. Its support is derived from a legacy left by Josiah Randall.

On Blackwell's Island is situated the *Penitentiary*, of which in 1852 the inmates numbered 5,323.

*The Penitentiary Hospital.*—The inmates in 1852 were 1,156 males, and 1,778 females.

*The Small Pox Hospital.*—There were treated in this institution in 1852, 102 males and 57 females.

*The Alms House.*—Total number of Inmates in 1852, was 1,624.

*The Lunatic Asylum.*—Whole number treated in 1852, 1,012. The number of inmates at this time is over 500.

These Institutions belong to the City, and are under the control and direction of the Governors of the Alms House. The members of the Association were cheered by a sumptuous repast given by the Ten Governors, Wm. West, the Chairman, representing the hosts and presiding. After being filled with the choicest fruits of the Island and having drunk a few bumpers, we again took the boat and bore on our winding way to the City, where in a few hours we were moving in every direction—homeward bound.

Very respectfully yours,

J. C. HUGHES.

